# Evaluation of the Biosynex AMPLIQUICK^®^ Fecal Parasitology assays in a Belgian travel clinic

**DOI:** 10.3389/fcimb.2026.1816447

**Published:** 2026-10-07

**Authors:** Jasmine Coppens, Jean-Marc Schwob, Karin Van Looveren, Jolien Baeyens, Marjan Van Esbroeck

**Affiliations:** 1 Department of Clinical Sciences, Institute of Tropical Medicine, Nationalestraat, Antwerp, Belgium; 2 Division of Tropical and Humanitarian Medicine, Geneva University Hospitals, Geneva, Switzerland; 3 Clinical Microbiology Laboratory, Geneva University Hospitals, Geneva, Switzerland

**Keywords:** helminths, microscopy, multiplex PCR, parasites, protozoa

## Abstract

**Introduction:**

Molecular diagnostics have improved infectious disease detection, yet their role in parasitology remains nuanced.

**Methods:**

We compared the performance of the AMPLIQUICK^®^Fecal Parasitology assay with the conventional methods encompassing microscopy, antigen testing and in-house PCR testing, in a reference laboratory for parasitology in a travel clinic. The detection of protozoa, helminths, and microsporidia was evaluated on 107 stool samples from 99 patients with suspected gastrointestinal parasitic infections, of which 40 specimens were tested retrospectively and 67 were collected prospectively.

**Results:**

AMPLIQUICK^®^ demonstrated high accuracy for protozoa and microsporidia (94.6%, 35/37) detecting all *Entamoeba histolytica*, *Entamoeba dispar*, most *Giardia duodenalis* and *Cryptosporidium* spp., and all *Enterocytozoon bieneusi* and *Encephalitozoon* spp. The assay detected more *Dientamoeba fragilis* (24 vs. 12) and *Blastocystis* sp. (61 vs. 44) than the conventional method but the correctness of these results could not be established. AMPLIQUICK^®^ reliably detected most clinically relevant helminths, including *Ascaris lumbricoides*, *Strongyloides* spp., *Hymenolepis nana*, *Taenia* spp., and *Enterobius vermicularis.* Hookworms, *Trichuris trichiura*, *Schistosoma mansoni*, *Heterophyidae/Opisthorchidae* and *Trichostrongylus* spp. were occasionally missed.

**Discussion:**

AMPLIQUICK^®^ demonstrated good performance compared to the conventional methods and has the potential to improve parasitology diagnostics, particularly in laboratories with limited expertise. Clinicians should interpret findings in the patient’s clinical context.

## Introduction

Molecular diagnostics have significantly improved the detection and control of infectious diseases, enabling more effective and faster treatment, resulting in lower morbidity and mortality rates among patients. Syndromic testing panels have revolutionised diagnostic procedures for diseases like bloodstream infections, gastroenteritis, and respiratory infections by simultaneously detecting a wide variety of pathogens (Dien Bard and McElvania, 2020). Conventional diagnostic techniques often require extended processing times, which may delay treatment, lengthen hospital stays, and raise healthcare expenses. While PCR is the leading diagnostic method for viral and bacterial infections due to its speed and sensitivity (Yamin et al., 2023*,* Artika et al., 2022), its role in parasitology is more nuanced. PCR offers improved sensitivity for protozoa, yet microscopy remains essential and is still the diagnostic gold standard for numerous parasitic infections (Coppens et al., 2024*,* Bradbury et al., 2022).

AMPLIQUICK^®^ Fecal Parasitology (Biosynex Illkirch, France) is a ready-to-use multiplex PCR platform for the detection of intestinal protozoa, microsporidia and helminths in stool samples, comprising a pretreatment assay (AMPLIQUICK^®^ Fecal Pretreatment) and two amplification assays (AMPLIQUICK^®^ Helminths and AMPLIQUICK^®^ Protozoans). A key advantage is its flexibility and compatibility with several in-house extraction systems and thermal cyclers.

This study aimed to evaluate the diagnostic performance of the AMPLIQUICK^®^ Fecal Parasitology assays by comparing them to the conventional diagnostic workflow, encompassing microscopy, antigen testing and in-house PCR testing, of the reference laboratory for parasitology of the Institute of Tropical Medicine (ITM).

## Methods

Study population: A total of 107 stool samples from 99 patients with suspected parasitic gastrointestinal illness were included in the study. Sixty-eight patients (with 76 samples) attended the ITM clinic, and stool samples from the remaining 31 patients, who attended other facilities, were sent to the ITM clinical laboratory in the scope of its reference function. Forty samples from the period 2012 to 2023 were selected from a collection of fresh frozen positive samples stored at -80 °C and tested retrospectively, while 67 fresh samples were prospectively collected during the patient’s work-out between January and December 2024, on the condition that sufficient material was available to perform all tests. Clinical and sociodemographic data were not available.

Multiplex PCR procedure: The stool samples were processed using the AMPLIQUICK^®^ Parasitology protocol, according to the manufacturer’s instructions, which comprises the AMPLIQUICK^®^ Fecal Pretreatment assay, and the two amplification assays, the AMPLIQUICK^®^ Protozoans and Helminths assay. In summary, 200 mg of stools and 1 ml of pretreatment buffer were bead-beat with grinding beads in a bead mill homogeniser (Bead Ruptor Elite, Omni), followed by 5 minutes incubation at 95 °C before centrifugation at 11000 g for 1 minute. This mix was stored at -20 °C until analysis, performed in batch once per month. DNA was extracted with the Maxwell^®^ RSC Blood DNA Kit on the Maxwell extraction automate (Promega, Wisconsin, United States). Real-time PCR with the AMPLIQUICK^®^ Protozoans and Helminths assays were performed on the Quantstudio 5 cycler (ThermoFisher, Massachusetts, United States; 0.2 ml tubes). The AMPLIQUICK^®^ Protozoans assay targets *Blastocystis* sp., *Cryptosporidium* sp., *Cyclospora cayetanensis*, *Cystoisospora belli*, *Dientamoeba fragilis*, *Entamoeba dispar*, *Entamoeba histolytica*, *Giardia duodenalis* and the microsporidia *Encephalitozoon* spp. and *Enterocytozoon bieneusi*. The AMPLIQUICK^®^ Helminths assay targets *Ancylostoma duodenale*, *Ascaris lumbricoides*, *Enterobius vermicularis*, *Necator americanus, Strongyloides* spp., *Trichuris trichiura, Dibotriocephalus latus*, *Hymenolepis nana, Taenia* sp. and *Schistosoma mansoni*. Testing of the samples was performed in 10 batches, spread over a period from April 2024 to December 2024.

The diagnostic procedure of the clinical laboratory of the ITM (further referred to as the conventional method, **Figure 1**) included microscopic examination of unstained and iodine-stained direct smears, wet mounts after formalin-ether concentration, iron hematoxylin Kinyoun staining of sodium acetate-acetic acid-formalin (SAF)-fixed stools, carbol-fuchsine staining on formalin-ether concentrates, the Baermann technique to detect *S. stercoralis* larvae, and copro-antigen enzyme-linked-immunosorbent-assays (ELISAs) for the detection of *Giardia*, *Cryptosporidium*, and *E. histolytica/E. dispar* (ProSpecT, OXOID Inc., Nepean, ON, Canada) depending on the request of the physician. Molecular methods included PCR to differentiate between *E. histolytica* and *E. dispar* performed on all samples positive for *E. histolytica/dispar* with microscopy or ELISA (Cnops and Van Esbroeck, 2010), PCR to detect *Strongyloides* spp. (in case the conditions to perform the Baermann concentration technique were not met*), E. bieneusi*, and *Encephalitozoon* species on the request of the physician, and PCR to detect *G. duodenalis* (adapted from Verweij et al., 2003*)*, and *Cryptosporidium hominis* and *Cryptosporidium parvum* (adapted from Hadfield et al., 2011) used in case of discrepancy between microscopy and ELISA (Hadfield et al., 2011), and is this study additionally performed to confirm discrepancies between AMPLIQUICK^®^ and the conventional method. All samples for PCR testing were frozen at -20 °C for at least 24 hours before analyses (Cnops and Van Esbroeck, 2010). Samples with weak positive PCR results (Ct value ≥ 38) were retested and considered negative if the second PCR result was negative. The detection of *E. vermicularis* eggs was performed on demand using the scotch-tape method.

**Figure 1:**
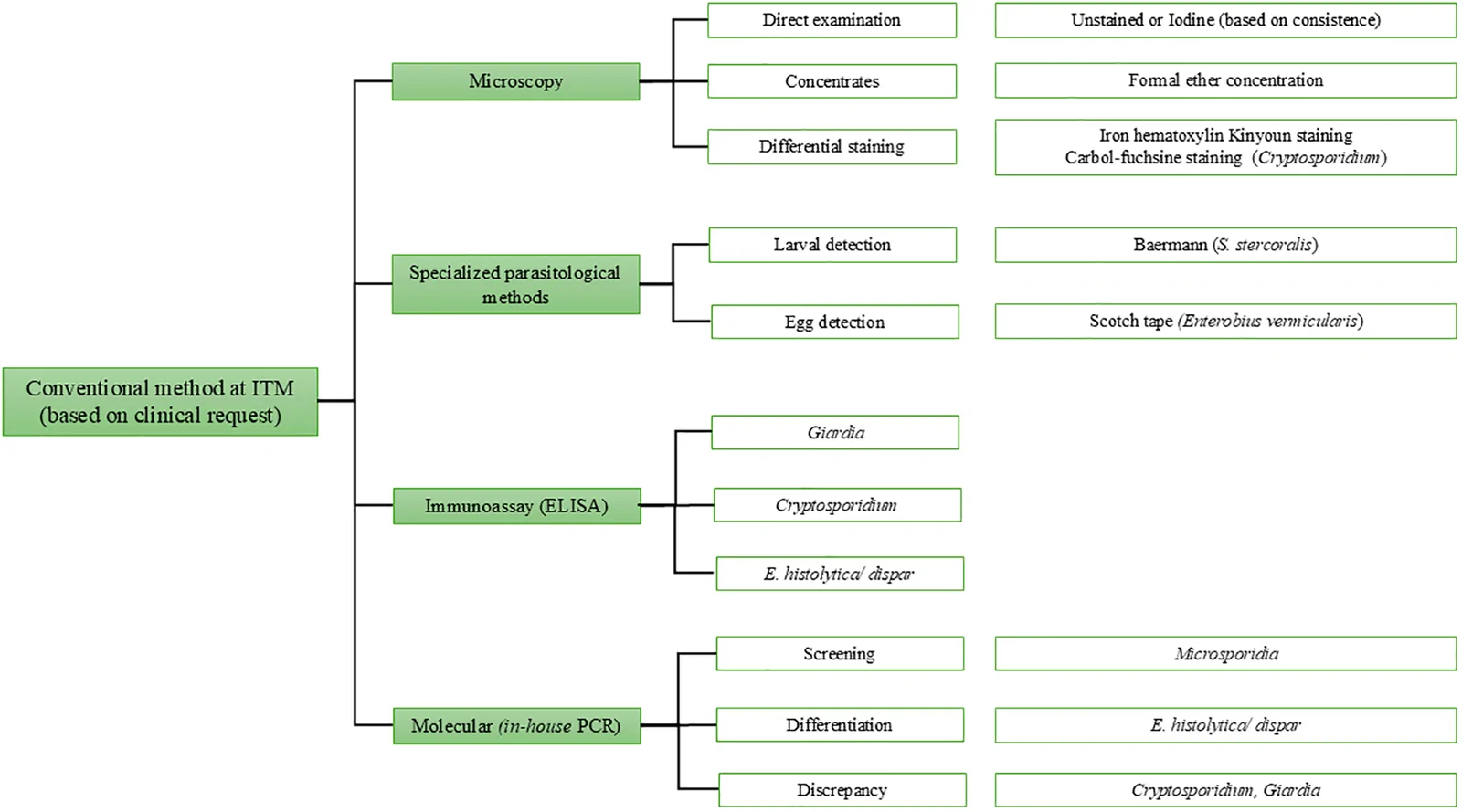
Flowchart of the conventional method at ITM including the different diagnostic methods used.

Samples in which helminths and protozoa were detected with the conventional method and not by AMPLIQUICK^®^ were considered as true positive. Findings with AMPLIQUICK^®^ and not with the conventional methods were confirmed by additional PCRs if available. Detections of pathogens, including *D. fragilis* and *Blastocystis* sp., detected only with AMPLIQUICK^®^, which could not be confirmed with additional methods, are described without strong conclusions on the performance of the assay under evaluation.

## Results

Of the 107 stool samples included in the study, 102 were positive by at least one method for one or more intestinal parasites or microsporidia with a total of 239 unique parasites and microsporidia detected. Using the conventional method, 101 samples tested positive, yielding a total of 200 parasites or microsporidia. AMPLIQUICK^®^ detected 188 unique parasites or microsporidia in 96 samples.

Both methods demonstrated a high accuracy for pathogenic protozoa, correctly identifying 35/37 (94.6%, 95% CI 81,8–99,3%) (**Table 1**). The AMPLIQUICK^®^ Protozoans kit detected all *E. histolytica* (5/5), *E. dispar* (26/26), *E. bieneusi* (3/3) and *Encephalitozoon* spp. (1/1), 18/19 *G. duodenalis* and 5/6 *Cryptosporidium*, positive with the conventional method (**Table 2**). The *G. duodenalis* sample missed by AMPLIQUICK^®^ was positive with the antigen detection ELISA and weakly positive by the in-house PCR, with only one of two replicates yielding a positive result (Ct value 40). The *Cryptosporidium* sample missed by AMPLIQUICK^®^ was weakly positive (Ct value 40), and was identified as *Cryptosporidium* sp. other than *C. hominis* or *C. parvum* based on the in-house PCR. AMPLIQUICK^®^ detected one additional *C. cayetanensis* and one *C. belli*, both with a high Ct value (34.6 and 36.8 respectively) which may explain the negative result of the conventional method. AMPLIQUICK^®^ detected more *D. fragilis* (24 vs. 12 cases) and *Blastocystis* sp. (61 vs 44 cases) than the conventional method. These extra findings were not confirmed with an additional method. Seven detections by AMPLIQUICK^®^ (1 *Cryptosporidium* spp., 4 *E. dispar*, and 2 *G. duodenalis*) were not detected by the conventional method during the initial patient’s workout because PCR was not performed but were retrospectively confirmed by the in-house PCR.

**Table 1:** Comparison of protozoa, microsporidia and helminth detections according to the diagnostic method.

Method	Protozoa/microsporidia		Pathogenic protozoa		Helminths	
	n	%	n	%	n	%
Conventional Method	160	82.1	35	94.6	40	90.9
AMPLIQUICK^®^ assay	150	76.9	35	94.6	38	86.4
Either conventional method or AMPLIQUICK^®^ assay	195		37		44	

**Table 2:** Sensitivity of the conventional method and AMPLIQUICK^®^ per species. ND, not determined.

Parasite			Positive samples with conventional method	Positive samples with AMPLIQUICK^®^	Positive samples with either method	Sensitivity ITM standard workflow (%)	Sensitivity AMPLIQUICK^®^ (%)
Included in AMPLIQUICK® panel	Helminths	*Ankylostomatidae*	8	7	9	88.9%	77.8%
		*Ascaris lumbricoides*	4	4	4	100%	100%
		*Enterobius vermicularis*	2	5	5	40%	100%
		*Strongyloides* spp.*	6^**^	6	6	100%	100%
		*Trichuris trichiura*	4	3	4	100%	75%
		*Dibotriocephalus latus*	0	0	0	/	/
		*Hymenolepis nana*	2	2	2	100%	100%
		*Taenia* spp.	6	6	6	100%	100%
		*Schistosoma mansoni*	6	5	6	100%	83.3%
	Fungi	*Encephalitozoon*	1	1	1	100%	100%
		*Enterocytozoon bieneusi*	3	3	3	100%	100%
	Protozoa	*Blastocystis* sp.	41	61	62	66.1%	98.4%
		*Cryptosporidium* spp.	6^**^	5	6	100%	83.3%
		*Cyclospora cayetanensis*	4	5	5	80%	100%
		*Cystoisospora belli*	1	2	2	50%	100%
		*Dientamoeba fragilis*	12	24	24	50%	100%
		*Entamoeba dispar*	26^**^	26	26	100%	100%
		*Entamoeba histolytica*	5	5	5	100%	100%
		*Giardia duodenalis*	19^**^	18	19	100%	94.7%
Not included in AMPLIQUICK® panel	Helminths	*Heterophyidae/Opisthorchidae*	1	ND	1	100%	ND
		*Trichostrongylus* spp.	1	ND	1	100%	ND
	Protozoa	*Chilomastix mesnili*	6	ND	6	100%	ND
		*Endolimax nana*	16	ND	16	100%	ND
		*Entamoeba species*	1	ND	1	100%	ND
		*Entamoeba coli*	8	ND	8	100%	ND
		*Entamoeba hartmanni*	7	ND	7	100%	ND
		*Iodamoeba butschlii*	4	ND	4	100%	ND

*S. stercoralis (n=5) and *S. fuelleborni* (n=1). **The conventional method (PCR) was only positive in retrospect for *Strongyloides* spp. *(n=3)*; *Cryptosporidium* (n=1); *E. dispar* (n=4) and *G. duodenalis* (n=2).

The AMPLIQUICK^®^ Helminths assay detected all *A. lumbricoides (4/4)*, *Strongyloides* spp. (6/6), *H. nana (2/2)* and *Taenia* spp. (6/6), positive with the conventional method. AMPLIQUICK^®^ failed to detect 1/9 hookworms and 1/4 *T. trichiura*, detected by microscopy and 1/6 *S. mansoni* detected by microscopy and in-house PCR (Ct value 32.7). The AMPLIQUICK^®^ Helminth kit detected five cases of *E. vermicularis* (Ct- values: 23.82-28.96) whereas the conventional workflow only detected two, one by direct examination of a stool sample and one after SAF fixation and iron hematoxylin Kinyoun staining. No scotch test had been performed. Three detections of *Strongyloides* spp.by AMPLIQUICK^®^ had not been detected by the conventional method because PCR was not performed during the patient’s workout, but only retrospectively to confirm the finding with AMPLIQUICK^®^.

The AMPLIQUICK^®^ assays did not detect two pathogenic helminths (1 *Heterophyidae*/*Opisthorchidae* and 1 *Trichostrongylus* spp.) and 42 non-pathogenic protozoa as the targets of these organisms were not included in the PCR multiplex panel.

## Discussion and conclusion

We compared the performance of AMPLIQUICK^®^ Fecal Parasitology assay to ITM’s conventional diagnostic workflow for the detection of protozoa, helminths, and the microsporidia *Encephalitozoon* spp. and *E. bieneusi*. Overall, AMPLIQUICK^®^ demonstrated strong performance across most parasite species.

The AMPLIQUICK^®^ Protozoans assay performed equally well as the conventional method for the detection of almost all pathogenic protozoa tested, *E. dispar*, and the microsporidia *Encephalitozoon* and *E. bieneusi*. The number of microsporidia in the study was however limited, since testing for microsporidia is rarely requested and only reimbursed in immunocompromised patients in Belgium.

The AMPLIQUICK^®^ assay missed 1/6 *Cryptosporidium* spp. and 1/19 *G. duodenalis* detected with the conventional method leading to sensitivities of 83,3% and 94,7% respectively. In comparison, reported sensitivities for other molecular assays targeting *G. duodenalis* are 89% for BD MAX™ (Becton Dickinson), 64% for G-Diapara™ (Diagenode Diagnostics), and 41% for RIDA^®^GENE (R-Biopharm) (Autier et al., 2018). The slightly lower sensitivity of 75% for *C. parvum/hominis* observed with BD MAX™ was mainly attributed to the presence of PCR inhibitors, resulting in 25% (2/8) false-negative results (Autier et al., 2018).

Additional findings such as the detection of one *C. belli* and one *C. cayetanensis* with AMPLIQUICK^®^ are not surprising and are consistent with prior studies on multiplex PCRs showing good performances in diagnosing protozoa (Coppens et al., 2024; Autier et al., 2020). Whether both pathogens were not detected by microscopy because of the low DNA concentration (high Ct-value) or were false-positive with AMPLIQUICK^®^ could however not be established.

Most discrepancies in favour of the conventional method concerned protozoans that were not included in the AMPLIQUICK^®^ panel. The conventional method therefore demonstrated logically superior performance for the detection of *Chilomastix mesnili, Endolimax nana, Entamoeba coli, Entamoeba hartmanni and Iodamoeba bütschlii.* Since these protozoans are considered non-pathogenic, their detection would not have had therapeutic consequences.

The AMPLIQUICK^®^ kit detected higher numbers of *D. fragilis* and *Blastocystis* sp. than the conventional method. Since these protozoans could only be found with the conventional method when the iron hematoxylin Kinyoun staining of SAF-fixed stools was performed on fresh stools (*D. fragilis*), an exam that had to be requested by the treating physician, or when fresh, unfixed stools were examined (*Blastocystis* sp.), it is possible that the AMPLIQUICK^®^ assay outperformed the conventional method. In the absence of a reference method, false-positive results of AMPLIQUICK^®^ could however not be excluded. The pathogenicity of *D. fragilis* and *Blastocystis* sp. remains debated and their detection would hardly have had therapeutic consequences.

The good performance of the AMPLIQUICK^®^ Helminths assay is to underline, with results closely matching the conventional method (38/44 vs. 40/44). The detection of *Ascaris lumbricoides, Hymenolepis nana, Strongyloides* spp. and *Taenia* spp. exhibited perfect concordance between the two methods although AMPLIQUICK^®^ did not differentiate *S. stercoralis* from *S. fuelleborni*. Small discrepancies were observed for *T. trichiura, Ankylostomatidae* and *S. mansoni*, for which AMPLIQUICK^®^ showed slightly lower detection rates than the conventional method. In one sample*, Trichostrongylus* eggs were found by the conventional method, while AMPLIQUICK^®^ detected *Strongyloides* spp. and *Necator*. The in-house PCR on the AMPLIQUICK^®^ DNA extract confirmed the presence of *Strongyloides* spp. Whether the target of AMPLIQUICK^®^ for *Necator* cross-reacts with *Trichostrongylus* could not be excluded. The AMPLIQUICK^®^ Helminths assay detected more *E. vermicularis* than the conventional method. In both the cases of the *Strongyloides* spp. and the three *E. vermicularis* not detected with the conventional method, the treating physician did not specifically request to look for these pathogens during the patient’s workout and the Baermann concentration or Strongyloides PCR, respectively the scotch-tape tests were therefore not performed. The extra finding of *Strongyloides* spp. in one sample illustrates a major advantage of multiplex PCRs to detect important pathogens not specifically thought of and asked for by the physician. The additional findings of *E. vermicularis* with AMPLIQUICK^®^ could not be confirmed by the conventional method in retrospect and false-positive results of AMPLIQUICK^®^ are therefore not excluded.

Treggiari et al. reported a sensitivity of 86.2% (25/29; 95% CI: 67.4–95.5%) for the detection of *Strongyloides* spp. with AMPLIQUICK^®^, compared to 89.7% (26/29, 95% CI: 72.6-97.8%) with an in-house RT-PCR. AMPLIQUICK^®^ additionally detected two *T. trichiura*, three *N. americanus* and one *Strongyloides* spp., while the in-house RT-PCR uniquely detected one *N. americanus* and one *S. mansoni* (Treggiari et al., 2025). These observations are in line with our findings. The sensitivity of 83.3% for *S. mansoni* in our study is better than the sensitivity of 59.1% (43.3–73.3%), 26/44) reported by Treggiari et al. for both AMPLIQUICK^®^ and the in-house RT-PCR *(*Treggiari et al., 2025).

For the detection of helminths, AMPLIQUICK^®^ outperformed SeeGene Allplex™ that detected a lower proportion of hookworms (2/3; 66.6%), *Ascaris* spp. (3/5; 60%), *E. vermicularis* (2/3; 66.6%), and *T. trichiura* (1/5; 20%) compared to the same conventional methods (Coppens et al., 2024). Of note, 22 samples tested with SeeGene Allplex™ by Coppens et al. in 2024 (Coppens et al., 2024), were also tested in this study with AMPLIQUICK^®^ with a better performance of the latter for *T. trichiura*, *S. mansoni*, *A. lumbricoides*, *Ankylostomidae* and *E. vermicularis* and an equal performance for *S. stercoralis*.

The better performance of the AMPLIQUICK^®^ assay to detect helminths compared to the other multiplex PCR test evaluated in our previous study (Coppens et al., 2024) and PCR tests described by others (Autier et al., 2020*;* Kaisar et al., 2017), is probably due to the improved sample preparation enhancing helminth’s eggs lysis and increasing the release of DNA. Most helminth’s eggs are highly resistant as their structure is adapted to protect larvae until conditions are optimal for lifecycle continuation (WHO, 2025) and this may hinder DNA release during the extraction procedure. It has been demonstrated before that standard bead-beating procedures with glass beads do probably not allow sufficient lysis of eggs and subsequent homogenization of the genetic material, in particular with eggs present in very small quantities (Coppens et al., 2024; Kaisar et al., 2017), although difference in bead-beating pretreatments exists (De Vivero et al.,2026). During the AMPLIQUICK^®^ Pretreatment step stools are mechanically grinded with abrasive garnet sand in the Ruptor Elite Bead Mill probably leading to better destruction of egg walls and hence increased homogenization of the parasitic material. In one entomology study comparing four different ruptors, the Ruptor Elite Bead Mill used by Biosynex performed best (Jones et al., 2020). It should be noted that all samples in our study were frozen after the Pre-Treatment step which is only one of two possible procedures prescribed by the manufacturer and may have had an additional positive effect on the sensitivity (Cnops and Van Esbroeck, 2010). Another factor that may have contributed to the good performance of the AMPLIQUICK^®^ in this study is the use of a calibrated amount of stool rather than a fecal swab, ensuring adequate sample input and allowing sampling from different parts of the stool specimen to minimize the risk of sampling errors.

Although the AMPLIQUICK^®^ panel includes most of the relevant helminths, some important hepatic trematodes are missing. *Fasciola* spp. with at least 2.4 million people infected (Fürst et al., 2012*)* would be a highly relevant additional target. The same is true for *Schistosoma* species other than *S. mansoni* such as *S. haematobium, S. japonicum* and *S. mekongi*, and *Schistosoma* hybrid species, although more rarely found in stools (De Elías-Escribano et al., 2025; Soentjens et al., 2016*).*

Other pathogens not included in the AMPLIQUICK^®^ panel are *Opisthorchis* spp. and *Clonorchis sinensis* that, although very prevalent in Cambodia (Yong et al., 2014) and South-East China (Lin et al., 2024) respectively, are very rarely reported in import medicine (Cociancic et al., 2025).

A limitation of our study is the small sample size for some samples. Although all parasites present in the AMPLIQUICK^®^ panel except *Dibotriocephalus latus* were included in the study, only a small number of samples positive for some parasites was analysed which precluded robust species-specific performance estimates. The small sample size did not allow stratification of the results by retrospective and prospective groups of samples. A second limitation is the fact that all samples were frozen after the Pre-Treatment step and analysed in batch. The alternative procedure prescribed by the manufacturer to test samples within 48 hours without freezing them, could therefore not be evaluated. Since freezing itself may increase the DNA yield, this difference may be important (Cnops and Van Esbroeck, 2010). A final limitation is the inability to confirm or rule out the additional detections of *D. fragilis, Blastocystis* sp.*, C. belli*, *C. cayetanensis*, hookworm and *E. vermicularis* detected by AMPLIQUICK^®^, preventing us from clearly determining whether these reflected increased sensitivity compared with the conventional method or false-positive results.

Despite the good diagnostic performance of multiplex PCR systems, with the major advantage to detect pathogens not specifically thought of and asked for by the physician, replacing a conventional workflow largely based on microscopy by a system uniquely based on molecular detection, raises important considerations. While multiplex PCRs can detect a wide range of pathogens, only pathogens included in the panel can be detected. The amount of stools analysed by PCR is often smaller than the amount examined by microscopy which may limit its sensitivity and may lead to sampling errors, especially in case of low parasitic density or inhomogeneous distribution in the stools lowering the chance of capturing the targeted pathogen in the aliquot examined. Finally, it is essential for clinicians to interpret results carefully, as not all findings may be clinically relevant or require therapeutic intervention, such as the detection of *Blastocystis* sp. and *D. fragilis* with questioned pathogenicity, or the finding of microsporidia.

In conclusion, the AMPLIQUICK^®^ performed well in diagnosing protozoa, microsporidia, and helminths, with sensitivities comparable to those of the conventional method. This multiplex PCR has the potential to improve parasite diagnostics in microbiology laboratories, particularly those with limited expertise. The large width of the diagnostic panel may lead to a substantial increase of findings. Since the panel covers not only pathogenic but also harmless parasites, a good understanding of the meaning of positive results by the clinician is needed.

## Data Availability

The raw data supporting the conclusions of this article will be made available by the authors, without undue reservation.
